# Combined maternal KIR2DL4 and fetal HLA-G polymorphisms were associated with preeclampsia in a Han Chinese population

**DOI:** 10.3389/fgene.2024.1442938

**Published:** 2024-07-31

**Authors:** Yantuanjin Ma, Yuan Qian, Hong Jiang, Haiyun Meng, Yang Wang, Yuling Yang

**Affiliations:** ^1^ College of Basic Medical Sciences, Kunming Medical University, Kunming, China; ^2^ Institute of Biomedical Engineering, Kunming Medical University, Kunming, China; ^3^ Prenatal Diagnosis Center, The first Affiliated Hospital of Kunming Medical University, Kunming, China; ^4^ Precision Medicine Center, The Affiliated Hospital of Yunnan University (The Second People’s Hospital of Yunnan Province), Kunming, China; ^5^ Clinical Laboratory, Jiangxi Province Hospital of Integrated Chinese and Western Medicine, Nanchang, China; ^6^ Obstetrics Department, Yan’an Hospital of Kunming City, Kunming, China; ^7^ Clinical Laboratory, Yan’an Hospital of Kunming City, Kunming, China

**Keywords:** preeclampsia, polymorphism, Placenta, HLA-G, KIR2DL4

## Abstract

Preeclampsia is the main cause of maternal and infant mortality and morbidity during pregnancy. Killer cell immunoglobulin-like receptor 2DL4 (KIR2DL4) and human leukocyte antigen G (HLA-G) play crucial roles in immune tolerance at the maternal-fetal interface. In this case‒control study, 154 maternal–fetal pairs were recruited, including 74 pairs with preeclampsia (56 of 74 pairs from family triads) and 80 pairs with a normal pregnancy (78 of 80 pairs from family triads). SNaPshot technology was used to detect genetic polymorphisms for 7 TagSNPs in the *KIR2DL4* and *HLA-G* genes. Among the fetal *HLA-G* gene polymorphisms, rs9380142 (A vs. G: OR = 2.802, 95% CI = 1.761–4.458) and rs1063320 (G vs. C: OR = 1.807, 95% CI = 1.144–2.852) differed between the preeclampsia group and the control group. The transmission disequilibrium test (TDT) suggested that the differences in the rs9380142G/A polymorphism in foetuses between preeclampsia triads and control triads were due to differences in transmission from the parents (*P* = 0.001). There was no significant difference in the distribution of maternal *KIR2DL4* alleles or genotype frequency between the preeclampsia group and the control group. Gene‒gene interaction analysis revealed that the combined genotypes of maternal rs649216-CC and fetal rs9380142-GG, maternal rs1051456-CG/GG and fetal rs9380142-GG, maternal rs34785252-CC and fetal rs9380142-AA/GA, and maternal rs34785252-CC/AA and fetal rs9380142-GG were associated with a significantly lower risk of preeclampsia. Therefore, this study suggested that the combination of maternal KIR2DL4 and fetal HLA-G polymorphisms was associated with preeclampsia in a Han Chinese population.

## 1 Introduction

Preeclampsia is a pregnancy-specific disorder characterized by hypertension and proteinuria after 20 weeks of gestation (Gestational Hypertension and Preeclampsia 2020). It is often accompanied by edema, fetal growth restriction, and other complications that pose serious health risks to both the mother and the fetus, and is a common obstetrical complication with an incidence of 5%–10% (Al-Jameil et al., 2014). According to statistics, preeclampsia causes 3.9% of maternal deaths and 10.7% of perinatal fetal deaths (Colbern, et al., 1994), seriously affecting the health of mothers and infants. Currently, most scholars believe that disruption of the immune balance at the maternal-fetal interface in the gestational state is the main cause of preeclampsia (Mukherjee, et al., 2023).

The maternal-fetal interface consists mainly of trophoblasts from the embryo and decidua from the mother. Placental trophoblasts do not express classical human leukocyte antigen (HLAIa) or HLAII but rather express nonclassical class I molecules, i.e., HLA-C, HLA-E and HLA-G (Colbern, et al., 1994; Ferreira, et al., 2017). The HLA-G gene is located on chromosome 6 and contains 8 exons and 7 introns. It has a strict tissue distribution and is expressed at high levels in extravillous trophoblasts (EVTs) (Ferreira, et al., 2017). Low expression and/or secretion of HLA-G in the placenta is associated with preeclampsia (Emmer, et al., 2004; Tang, et al., 2015). The HLA-G gene is polymorphic, particularly in the 3′-untranslated region (UTR), which contains several regulatory elements, including AU-rich elements, polyadenosine signals and signals regulating mRNA spatial and temporal expression (Reches, et al., 2020). HLA-G gene polymorphisms may primarily affect the stability of HLA-G mRNA, which in turn affects HLA-G expression. HLA-G exists in both soluble and membrane-bound forms, and its interactions with maternal immune cells play a critical role in maintaining immune tolerance during pregnancy (Mao, et al., 2023).

The decidua of early pregnancy has a large number of immune cells (Kaminski, et al., 2019), 70% of which are uterine natural killer (uNK) cells (Parham, 2004), which have low cytotoxicity (Moffett and Shreeve, 2015). uNK cells express killer cell immunoglobulin-like receptors (KIRs) on their surface. The extracellular domain of the KIR protein binds to its ligand, the HLA-I molecule. The KIR gene family is located on chromosome 19 and consists of 15 genes and 2 pseudogenes (Ritari, et al., 2022). KIR gene polymorphisms are closely related to their expression and the functional activity of their products. KIR2DL4, a member of the KIR family, is expressed on the surface of all NK cells, and its ligand is HLA-G (Colucci, 2017).

EVT cells are highly invasive uterus cells that mediate the remodeling of the uterine spiral arteries to ensure the normal exchange of material between the mother and foetus, which is essential for normal embryonic development. EVT invasion is regulated mainly by uNK cells (Kieckbusch, et al., 2014; Colucci, 2017). Maternal KIRs bind to fetal HLAs and regulate uNK cell function through the KIR-HLA receptor‒ligand pathway (Kieckbusch, et al., 2014; Colucci, 2017). When abnormal uNK cell function leads to insufficient EVT invasion into the uterus, the placenta is poorly formed, triggering preeclampsia (Hiby, et al., 2004; Nakimuli, et al., 2015). Thus, maternal and fetal factors play an equally important role in the immune imbalance between uNK cells and EVTs that triggers preeclampsia. Most of the current studies only consider genetic factors, molecular expression or associated microenvironmental changes in mothers, ignoring the influence of fetal factors. In this study, we investigated the polymorphisms of maternal KIR2DL4 and fetal HLA-G genes and assessed the correlation between different maternal-fetal genotype combinations and preeclampsia to identify preeclampsia susceptibility genes and to aid in the early diagnosis and prediction of this disease.

## 2 Materials and methods

### 2.1 Subjects

In this case‒control study, 154 maternal–fetal pairs were recruited, including 74 pairs with preeclampsia (56 of 74 pairs from family triads) and 80 pairs with a normal pregnancy (78 of 80 pairs from family triads). All samples were collected from the outpatient department of prenatal diagnosis at the First Affiliated Hospital of Kunming Medical University and Yan’an Hospital of Kunming city. These samples were used for preeclampsia evaluation. All participants were enrolled between September 2020 and June 2023. Preeclampsia was diagnosed using the criteria of the International Association for Pregnancy-Induced Hypertension (ISSHP). The diagnostic criteria were as follows: systolic blood pressure (SBP) and diastolic blood pressure (DBP) ≥140/90 mmHg present after 20 weeks of gestation, measured at least twice or at least 4 h apart, and albuminuria ≥300 mg/24 h. The control group was from the same center and consisted of pregnant volunteer women with normal blood pressure, and no history of preeclampsia. The exclusion criteria were metabolic abnormalities, chronic cardiovascular diseases, liver, kidney and endocrine diseases, Rh blood group incompatibility, autoimmune diseases and chromosome abnormalities. All participants signed an informed consent form for the collection of maternal peripheral blood and fetal umbilical vein blood samples. The study was approved by the Medical Ethics Committee of Kunming Medical University (Approval No. KMMU2020MEC027).

### 2.2 Sample collection and DNA extraction

During the 20th week of pregnancy, prenatal amniocentesis was performed by a professional obstetrician. In order to minimize risks to the mother and fetus, amniotic fluid is collected under sterile conditions and with ultrasound guidance. This procedure collected amniotic fluid for genetic analysis and scientific research. Peripheral blood samples were collected from parents for genotyping purposes. According to the manufacturer’s instructions, DNA was extracted from peripheral blood using the DNeasy Blood and Tissue Kit (QIAGEN, Germany). The obtained DNA was stored in a freezer at −20°C until analysis.

### 2.3 TagSNP selection and analysis

The necessary genotype data were obtained from the 1,000 Genomes Browser (https://www.ncbi.nlm.nih.gov/variation/tools/1000genomes/). TagSNPs were selected using HaploView 4.2 software with the following settings: upstream and downstream, 2K; minor allele frequency (MAF) ≥0.05; and linkage disequilibrium threshold (R2) ≥0.8. Three TagSNPs for the KIR2DL4 gene and four TagSNPs for the HLA-G gene were identified (Supplementary Table S1). The SNaPshot method was employed to analyze SNP polymorphisms. Details of the primers used are provided in Supplementary Table S2.

### 2.4 Statistical analysis

SPSS.229 (IBM) was used for the statistical analysis. For continuous variables, the Shapiro‒Wilk test was used for the normality test. Normally distributed continuous variables were expressed as the mean ± standard deviation, and nonnormally distributed variables were expressed as the median and quartile range (IQR). Classification variables were expressed as numbers or percentages. For each SNP, Pearson’s goodness-of-fit (χ2) statistic was utilized to evaluate the Hardy‒Weinberg equilibrium. The chi-square test or Fisher’s exact test was used to compare gene distributions among groups, and multivariate logistic regression was employed to calculate odds ratios (ORs) and 95% confidence intervals (CIs). The allelic associations between the preeclampsia group and the control group was evaluated. Taking advantage of the triad data, the transmission/disequilibrium test (TDT) statistic was calculated as follows: TDT χ^2^ = (a − b)^2^/(a + b). The *p*-value was calculated from a χ^2^ table with 1 df. Here, a was the number of times the interest allele is passed, and b was the number of times it has not been passed on. *p* < 0.05 was considered to indicate statistical significance.

## 3 Results

### 3.1 Clinical characteristics and SNP information

As shown in Table 1, no significant differences were observed between the two groups in age, BMI, previous misunderstandings, cesarean section and newborn sex (*p* > 0.05). However, there were statistical differences in gestational age, primiparity, parity, neonatal birth weight and neonatal length between the two groups (*p* < 0.05).

**TABLE 1:** Clinical characteristics of preeclampsia group and control group.

Variable	Preeclampsia	Control	*p*
Mother			
Age (years)	34.19 ± 3.86	33.31 ± 5.16	0.052
BMI (kg/m2)	23.9 ± 3.7	24.2 ± 2.9	0.574
Gestational age (weeks)	38.51 ± 2.52	37.66 ± 1.92	0.019
Primiparity (%)	58.10	37.50	0.015
Parity (times)	1.14 ± 0.27	1.97 ± 0.79	<0.001
Previous miscarriages (%)	29.72	28.75	0.919
Caesarian section (%)	39.19	38.75	0.991
Offspring			
weight (g)	2,854.10 ± 711.92	3,271.62 ± 431.55	<0.001
length (cm)	48.04 ± 3.57	49.27 ± 3.49	0.032
Male (%)	59.46	56.25	0.427

In this study, we tested 3 SNPs (rs649216, rs1051456, and rs34785252) for the maternal KIR2DL4 gene and 4 SNPs (rs9380142, rs1063320, rs1130363, and rs1630185) for the HLA-G gene in all family triads. The genotype distribution of all 7 SNP loci in the control group was consistent with HWE (Supplementary Table S3).

### 3.2 Allele frequencies in the preeclampsia group and control group

Among the four SNP loci in the fetal HLA-G gene, the frequencies of rs9380142A (OR = 2.802, 95% CI = 1.761∼4.458, *p* = 0.001) and rs1063320G (OR = 1.807, 95% CI = 1.144∼2.852, *p* = 0.01) were greater in the preeclampsia group than in the control group. No significant differences in allele frequency for the other loci in the fetal HLA-G gene were observed between the two groups.

Among the four SNP loci in the paternal HLA-G gene, the frequencies of the rs1630185G (OR = 1.644, 95% CI = 1.005∼2.688, *p* = 0.046) and rs1130363A (OR = 1.644, 95% CI = 1.005∼2.688, *p* = 0.046) alleles were lower in the preeclampsia group than in the control group. Allele frequencies at other loci of the paternal HLA-G gene were not significantly different between the two groups.

Maternal allele frequencies for all SNP loci of the KIR2DL4 gene and HLA-G gene were not significantly different between the preeclampsia group and the control group. See Table 2.

**TABLE 2:** Distributions of allele frequencies in the preeclampsia group and the control group.

SNPs	Gene	Allele	Minor allele	Minor allele preeclampsia	Minor allele control	OR (95%CI)	*X* ^*2*^	*p*
Mother								
rs649216	KIR2DL4	C/T	T	23 (0.155)	31 (0.193)	0.765 (0.423∼1.385)	0.781	0.376
rs1051456	KIR2DL4	G/C	G	90 (0.608)	80 (0.500)	1.551 (0.986∼2.44)	2.854	0.056
rs34785252	KIR2DL4	C/A	A	62 (0.418)	75 (0.468)	0.817 (0.52∼1.282)	0.773	0.379
rs9380142	HLA-G	G/A	A	61 (0.412)	80 (0.5)	1.426 (0.908∼2.238)	2.389	0.122
rs1063320	HLA-G	C/G	G	53 (0.358)	60 (0.375)	0.929 (0.584∼1.478)	0.094	0.758
rs1630185	HLA-G	A/G	G	72 (0.486)	66 (0.412)	1.349 (0.859∼2.117)	1.701	0.192
rs1130363	HLA-G	G/A	A	73 (0.493)	66 (0.412)	1.386 (0.883∼2.175)	2.024	0.154
Father								
rs9380142	HLA-G	G/A	A	45 (0.401)	52 (0.333)	1.389 (0.853∼2.262)	1.322	0.25
rs1063320	HLA-G	C/G	G	48 (0.428)	72 (0.461)	0.875 (0.536∼1.426)	0.286	0.592
**rs1630185**	HLA-G	A/G	G	56 (0.500)	97 (0.621)	1.644 (1.005∼2.688)	3.947	**0.046**
**rs1130363**	HLA-G	G/A	A	56 (0.500)	97 (0.621)	1.644 (1.005∼2.688)	3.947	**0.046**
Offspring								
**rs9380142**	HLA-G	G/A	A	85 (0.574)	62 (0.387)	2.802 (1.761∼4.458)	19.352	**0.001**
**rs1063320**	HLA-G	C/G	G	74 (0.5)	57 (0.356)	1.807 (1.144∼2.852)	6.499	**0.010**
rs1630185	HLA-G	A/G	G	61 (0.412)	75 (0.468)	0.794 (0.506∼1.247)	0.998	0.317
rs1130363	HLA-G	G/A	A	62 (0.418)	68 (0.425)	0.975 (0.620∼1.533)	0.011	0.914

The meaning of bold is: *p* < 0.05, which is statistically significant.

### 3.3 Genotype frequencies in the preeclampsia group and control group

For fetal rs9380142 in the HLA-G gene, the alleles are A and G, and the A allele is the minor allele. The GG, GA and AA genotype frequencies of the preeclampsia group were 21.6%, 41.8%, 36.4%, and 0.00%, respectively, and those of the control group were 42.5%, 37.5%, and 20.0%, respectively (*p* = 0.01). The A allele, which has a lower frequency in the population, is assumed to be a mutant gene. In the dominant mode, mothers of fetuses carrying this mutation were more likely to develop preeclampsia (AA + GA vs. GG, OR = 2.196, 95% CI = 1.009–4.780; *P* = 0.047). Under the recessive mode, this difference was not significant (AA vs. GA + GG). Therefore, we suggest that if the fetal A allele is associated with the development of preeclampsia in the mother, it may function in the dominant mode.

For fetal rs1063320 in the HLA-G gene, the alleles are G and C; the G allele is the minor allele. The CC, CG and GG genotype frequencies were 25.6%, 48.6%, and 25.6%, respectively, in the preeclampsia group and 46.2%, 35.2%, and 17.5%, respectively, in the control group (*p* = 0.029). The G allele, which has a lower frequency in the population, is assumed to be a mutant gene. Under the dominant mode, mothers of fetuses carrying this mutation were more likely to develop preeclampsia (GG + GC vs. CC, OR = 2.491, 95% CI = 1.259–4.927; *p* = 0.009). Under the recessive mode, this difference was not significant (GG vs. GC + CC). Therefore, we suggest that if the fetal G allele is associated with the development of preeclampsia in the mother, it may function in the dominant mode.

The genotype frequencies of rs1630185 and rs1130363 in the paternal HLA-G gene were both significantly different between the preeclampsia group and the control group (*p* = 0.01 and *p* = 0.01). They were all inherited in a recessive model in the population. No significant differences in genotype frequencies for the remaining detected SNPs in fetuses, fathers or mothers were observed between the two groups. See Table 3.

**TABLE 3:** Distributions of genotype frequencies in the preeclampsia group and the control group.

SNPs	Preeclampsia (n = 74)				Control (n = 80)				*p*	AB + BB vs. AA		BB vs. AA + AB	
	AA	AB	BB	Total	AA	AB	BB	Total		OR (95% CI)	*p*	OR (95% CI)	*p*
Mathor													
rs649216	53 (0.716)	19 (0.256)	2 (0.027)	74	53 (0.662)	23 (0.287)	4 (0.050)	80	0.725	0.778 (0.392∼1.544)	0.473	0.528 (0.094∼2.970)	0.468
rs1051456	13 (0.175)	32 (0.432)	29 (0.391)	74	23 (0.287)	34 (0.425)	23 (0.287)	80	0.196	0.626 (0.320∼1.227)	0.172	0.528 (0.245∼1.141)	0.104
rs34785252	25 (0.337	36 (0.486)	13 (0.175)	74	26 (0.325)	33 (0.412)	21 (0.262)	80	0.406	0.944 (0.482∼1.847)	0.866	0.599 (0.599∼1.305)	0.197
rs9380142	31 (0.418)	25 (0.337)	18 (0.243)	74	24 (0.300)	32 (0.400)	24 (0.300)	80	0.304	0.594 (0.306∼1.156)	0.125	0.430 (0.367∼1.533)	0.43
rs1063320	37 (0.5)	21 (0.283)	16 (0.216)	74	34 (0.425)	32 (0.4)	14 (0.175)	80	0.314	0.739 (0.391∼1.396)	0.351	1.300 (0.585∼2.892)	0.519
rs1630185	18 (0.243)	40 (0.54)	16 (0.216)	74	27 (0.337)	40 (0.5)	13 (0.162)	80	0.39	1.585 (0.783∼3.207)	0.2	1.422 (0.631∼3.202)	0.396
rs1130363	18 (0.243)	39 (0.527)	17 (0.229)	74	27 (0.337)	40 (0.5)	13 (0.162)	80	0.347	1.585 (0.783∼3.207)	0.2	1.537 (0.688∼3.434)	0.295
Father													
rs9380142	25 (0.446)	17 (0.303)	14 (0.25)	56	37 (0.474)	30 (0.384)	11 (0.141)	78	0.254	1.119 (0.562∼2.229)	0.749	2.030 (0.843∼4.889)	0.114
rs1063320	26 (0.464)	12 (0.214)	18 (0.321)	56	27 (0.346)	30 (0.384)	21 (0.269)	78	0.106	1.182 (0.571∼2.446)	0.652	1.464 (0.729∼2.942)	0.284
**rs1630185**	18 (0.321)	20 (0.357)	18 (0.321)	56	28 (0.358)	41 (0.525)	9 (0.115)	78	**0.010**	1.182 (0.571∼2.446)	0.652	3.632 (1.487∼8.868)	**0.005**
**rs1130363**	18 (0.321)	20 (0.357)	18 (0.321)	56	28 (0.358)	41 (0.525)	9 (0.115)	78	**0.010**	1.182 (0.571∼2.446)	0.652	3.632 (1.487∼8.868)	**0.005**
Offspring													
**rs9380142**	16 (0.216)	31 (0.418)	27 (0.364)	74	34 (0.425)	30 (0.375)	16 (0.200)	80	**0.010**	2.196 (1.009∼4.780)	**0.047**	1.633 (0.736∼3.622)	0.227
**rs1063320**	19 (0.256)	36 (0.486)	19 (0.256)	74	37 (0.462)	29 (0.362)	14 (0.175)	80	**0.029**	2.491 (1.259∼4.927)	**0.009**	1.629 (0.748∼3.544)	0.219
rs1630185	24 (0.324)	39 (0.527)	11 (0.148)	74	20 (0.250)	45 (0.562)	15 (0.187)	80	0.555	0.694 (0.344∼1.401)	0.309	0.757 (0.323∼1.773)	0.521
rs1130363	22 (0.297)	42 (0.567)	10 (0.135)	74	27 (0.337)	38 (0.475)	15 (0.187)	80	0.477	1.204 (0.610∼2.378)	0.593	0.677 (0.283∼1.618)	0.38

A: major allele, B: minor allele. The meaning of bold is: *p* < 0.05, which is statistically significant.

### 3.4 Transmission of rs9380142A and rs1063320G in family triads

To further examine the preeclampsia-associated SNP loci in fetuses identified in this case‒control study and to exclude false-positive results due to factors such as population stratification, we analyzed the rs9380142 and rs1063320 loci by using the transmission disequilibrium test (TDT). It should be noted that TDT analysis is not usually performed in a control group, but since this study had a case‒control design, we believe that performing TDT analyses in the control triads, with the help of opposite trends of transmission, would strengthen the results found in the case triads. The offspring were not the patients in our study, but the mothers were. Based on these considerations, we performed TDT in both the preeclampsia and control triads.

The genotype distributions of the rs9380142 and rs1063320 loci within preeclampsia and control triads are shown in Supplementary Table S4. TDTs were performed when parent-of-origin was not considered or when paternal or maternal transmissions were considered separately. As shown in Table 4, while no significant results were observed in the preeclampsia triad, in the control triad, the rs9380142A allele was insufficiently transmitted (less frequently inherited from fathers and mothers to their offspring). Only 4 of the 18 heterozygous fathers transmitted the rs9380142A allele to their offspring (*p* = 0.018; *X* ^*2*^ = 5.556, df = 1). Similarly, only 4 of 18 heterozygous mothers transmitted the rs9380142A allele (*P* = 0.018; *X* ^*2*^ = 5.556, df = 1). TDT revealed that differences in the rs9380142G/A polymorphism in offspring between the preeclampsia group and the control group were associated with differential parental transmission (*p* = 0.001). The significantly insufficient transmission of the rs9380142A allele in the control group reinforces the results of the present study that rs9380142A is a preeclampsia-associated allele (the risk allele). There were no significant differences in the transmission of the rs1063320G allele from heterozygous fathers or mothers to their offspring in the preeclampsia and control triads.

**TABLE 4:** Transmission disequilibrium test analysis of SNPs in HLA-G gene in family triads.

SNP	Allele		*Χ* ^*2*^, df 1	*p*-value
rs9380142	G	A		
Preeclampsia				
Transmitted from heterozygous parent	19	10	2.793	0.095
Transmitted from heterozygous mother	12	5	2.882	0.09
Transmitted from heterozygous father	5	3	0.5	0.48
Control				
Transmitted from heterozygous parent	28	8	11.111	**0.001**
Transmitted from heterozygous mother	14	4	5.556	**0.018**
Transmitted from heterozygous father	14	4	5.556	**0.018**
**rs1063320**	**C**	**G**		
Preeclampsia				
Transmitted from heterozygous parent	14	24	2.632	0.105
Transmitted from heterozygous mother	6	12	2	0.157
Transmitted from heterozygous father	6	10	1	0.317
Control				
Transmitted from heterozygous parent	29	28	0.018	0.895
Transmitted from heterozygous mother	11	8	0.474	0.491
Transmitted from heterozygous father	14	16	0.133	0.715

The meaning of bold is: *p* < 0.05, which is statistically significant.

### 3.5 Association of combined maternal KIR2DL4 and fetal HLA-G gene polymorphisms with preeclampsia

Maternal KIRs bind to fetal HLAs and regulate uNK cell function through the KIR-HLA receptor‒ligand pathway, which in turn affects placenta formation. Therefore, correlations between different maternal-fetal gene polymorphism combinations and preeclampsia were evaluated. The allele that has a lower frequency in the population is assumed to be a mutant allele. The entire SNP locus studied in the KIR2DL4 gene did not differ significantly between the two groups. The mothers were divided into mutant allele-containing and mutant allele-free groups. The TDT results indicated significant differences in allele transmission patterns between the preeclampsia group and the control group. Compared to that in preeclampsia triads, the transmission of the rs9380142A allele from parents to offspring is less frequent in the control triads. This finding suggested that the rs9380142A allele may increase the risk of preeclampsia by altering familial transmission dynamics, indicating that only rs9380142 in offspring is associated with the development of preeclampsia. rs9380142A in the HLA-G gene in offspring was inherited in a dominant mode in the study population. Therefore, the offspring were divided into two groups, i.e., minor allele-containing (AA + AG) and minor allele-free (GG) groups.

One-way ANOVA revealed that the genotype combinations of the three studied maternal KIR2DL4 gene SNP loci and fetal HLA-G gene rs9380142 were significantly different between the two groups (*p* < 0.05) (Table 5).

**TABLE 5:** The analysis of genotype combinations of maternal KIR2DL4 and fetal HLA-G.

Mother	Offspring	Preeclampsia	Control	*X* ^*2*^	*p*	Multiple logistic regression		
						OR	95% CI	*p*
**rs649216**	**rs9380142**							
CC	AA/GA	43	27	11.308	0.010	Ref		
CC	GG	10	26			0.242	0.101–0.579	**0.001**
CT/TT	AA/GA	15	19			0.496	0.216–1.138	0.098
CT/TT	GG	6	8			0.471	0.147–1.506	0.204
**rs1051456**	**rs9380142**							
CC	AA/GA	9	15	11.825	0.008	0.38	0.148–0.973	0.054
CC	GG	4	8			0.316	0.088–1.140	0.078
GC/GG	AA/GA	49	31			Ref		
GC/GG	GG	12	26			0.292	0.120–0.662	**0.003**
**rs34785252**	**rs9380142**							
CC	AA/GA	9	16	12.807	0.005	0.344	0.135–0.877	**0.025**
CC	GG	4	8			0.306	0.085–1.105	0.071
CA/AA	AA/GA	49	30			Ref		
CA/AA	GG	12	26			0.283	0.124–0.642	**0.003**

The meaning of bold is: *p* < 0.05, which is statistically significant.

Multiple logistic regression analyses were performed using the maternal KIR2DL4-fetal HLA-G genotype combinations that differed between the two groups via one-way ANOVA as the independent variable, preeclampsia as the dependent variable, and the genotype combination with the highest incidence as the reference group (Table 5). The results showed that for maternal rs649216-fetal rs9380142, the CC-GG genotype combination had a significantly lower risk of morbidity than the reference (*OR* = 0.242, *95% CI* = 0.101–0.579, *p* = 0.001), whereas the remaining two combinations were not significantly different from the reference. For maternal rs1051456-fetal rs9380142, the GC/GG-GG genotype combination had a significantly lower risk of morbidity than the reference (*OR* = 0.292, *95% CI* = 0.120–0.662, *p* = 0.003), whereas the remaining two combinations were not significantly different from the reference. For maternal rs34785252-fetal rs9380142, the CC-AA/GA genotype combination and CA/AA-GG genotype combination both had a significantly lower risk of morbidity than the reference (*OR* = 0.344, *95% CI* = 0.135–0.877, *P* = 0.025; *OR* = 0.283, *95% CI* = 0.124–0.642, *p* = 0.003), whereas the risk of morbidity for the remaining combinations was not significantly different from that of the reference.

## 4 Discussion

KIR2DL4 is a special member of the killer cell immunoglobulin-like receptor (KIR) family that can inhibit or activate NK cells through its different N-terminal structures. It is structurally expressed in all NK cells (Colbern, et al., 1994; Goodridge, et al., 2007) and is also highly expressed in decidual NK cells and placental NK cells (Brusilovsky, et al., 2013). KIR2DL4 activates resting NK cells to produce cytokines without causing cytotoxicity (Rajagopalan, et al., 2001). The production of cytokines such as tumor necrosis factor alpha (TNF-α), interleukin-1β (IL-1β) and interferon-γ (IFN-γ) induces the production of vascular endothelial growth factor (VEGF) by trophoblast cells, which in turn affects uterine angiogenesis (Brusilovsky, et al., 2013). At present, there are few reports on the relationship between KIR2DL4 polymorphisms and preeclampsia (Tan, et al., 2009). Our study revealed no significant differences in the genotype frequencies or allele frequencies of the three SNP loci in the KIR2DL4 gene between mothers in the preeclampsia group and mothers in the control group. In case‒control studies in Malaysia and Australia, no association between maternal KIR2DL4 allele frequency and preeclampsia was observed (Witt, et al., 2002; Tan, et al., 2009). This is consistent with the results observed in our study. Therefore, we concluded that the maternal KIR2DL4 gene is not strongly associated with the development of preeclampsia if it is studied as an independent genetic factor.

During placental implantation, EVT damages the arterial wall as it approaches the uterine spiral arteries through the decidua, allowing the fetus to receive an adequate supply of blood from the mother (Pollheimer, et al., 2018). Unlike other cells, EVT cells express three HLA class I molecules, HLA-C, HLA-E, and HLA-G. A high level of HLA-G expression is unique to EVT cells (Papuchova, et al., 2020). Many scholars believe that HLA-G molecules play an important role in maternal-fetal immunotolerance. The expression of HLA-G in the placenta is associated with preeclampsia (Colbern, et al., 1994; Goldman-Wohl, et al., 2000; Luo, et al., 2018). It has been shown that HLA-G is absent or has reduced expression levels in the placenta of most patients with preeclampsia (Goldman-Wohl, et al., 2000). Our results showed that both the allele frequencies and genotype frequencies of rs9380142 and rs1063320 in fetuses were significantly different between the two groups. Both rs9380142 and rs1063320 are located in the 3′UTR of the gene and are associated with mRNA localization, mRNA stability and protein translation regulation. In the study fetal population, the rs9380142A and rs1063320G allele frequencies (MAFs) were significantly greater in the preeclampsia group than in the control group, and the ORs were greater than 1, suggesting that these loci may be risk factors for disease development in the fetus.

Further TDT analyses revealed no significant difference in the frequency of transmission of the rs1063320G allele from the heterozygote parent to the offspring between the preeclampsia group and the control group. We therefore believe that the association of this SNP with disease was likely to be a false-positive result due to factors such as population stratification and should be excluded from subsequent analysis. TDT also showed that there was a significant transmission deficit of the rs9380142A allele in the control group, with both heterozygous parents being much less likely to transmit A to their offspring. Since the fetal rs9380142A allele was a risk factor for preeclampsia, its undertransmission in controls was in line with expectations. The findings of the TDT reinforce the results of the present study that rs9380142A is a risk allele in fetuses. Therefore, we suggest that fetal HLA-G gene polymorphisms are associated with the development of preeclampsia and are risk factors for disease development. Mutations may disrupt maternal-fetal immune tolerance and ultimately increase the risk of disease development by decreasing mRNA stability and downregulating HLA-G protein expression (Michita, et al., 2018).

At the time of embryo implantation and placenta formation, maternal uNK cells synergize with fetal EVTs, allowing EVTs to invade the uterus to clear and replace the smooth muscle of maternal spiral arterioles and allowing the recasting of small uterine spiral arterioles to nourish the growing fetus (Wei, et al., 2022). Interactions between the uNK cell receptors and their ligands on EVTs must occur to strike a delicate balance between overinvasion, which damages the mother, and underinvasion, which is insufficient to supply blood and oxygen to the fetus (Pollheimer, et al., 2018). Hiby et al. (2004) reported that the maternal KIR AA genotype and fetal HLA-C2 allotype are more common in the preeclampsia group, indicating that when the fetus carries the HLA-C2 allotype, mothers who lack most or all activating KIRs have a significantly increased risk of developing preeclampsia. Another finding showed that the direct interaction between HLA-G (+) breast cancer cells and KIR2DL4 (+) tissue mast cells was positively correlated with lymph node metastasis or lymphatic invasion in clinical specimens (Riteau, et al., 2001). These findings suggest that maternal-fetal-specific gene combinations mediating the interaction of uNK cells with EVTs are strongly correlated with disease development. Our study revealed four KIR2DL4/HLA-G genotype combinations that were associated with the development of preeclampsia and significantly reduced the risk of the disease in mothers. Interestingly, in all four genotype combinations, the fetus carried the rs9380142G allele in the HLA-G gene. The role of fetal HLA-G in maternal-fetal tolerance has been well established (Lynge Nilsson, et al., 2014), and this topic has been reported in many studies (Loisel, et al., 2013; Hsu, et al., 2014). Combined with our findings, we believe that fetal HLA-G contributes more to the establishment of maternal-fetal tolerance and therefore may be more relevant to the pathological mechanisms of preeclampsia. However, we did not analyze HLA-G expression in placental tissue. This may be a limitation of this study. On the other hand, although we observed significant associations between certain maternal KIR2DL4 and fetal HLA-G polymorphisms and preeclampsia, these findings need to be validated in larger and more diverse populations. Additionally, we did not consider potential environmental and lifestyle factors that might influence the development of preeclampsia. Future studies should incorporate comprehensive genomic approaches and consider the interactions between genetic, environmental, and lifestyle factors.

In conclusion, our findings underscore significant HLA-G gene polymorphisms in fetuses within the Chinese Han population, implicating fetal HLA-G as pivotal in preeclampsia pathogenesis. Despite the lack of a direct association between maternal KIR gene polymorphisms and preeclampsia, specific maternal KIR2DL4 and fetal HLA-G combinations warrant further exploration. This study contributes crucial insights into maternal-fetal immune tolerance mechanisms, guiding future genetic and clinical studies on preeclampsia.

## Data Availability

The original contributions presented in the study are included in the article/Supplementary Material, further inquiries can be directed to the corresponding authors.
