# Supplementary material for: Combined maternal KIR2DL4 and fetal HLA-G polymorphisms were associated with preeclampsia in a Han Chinese population
Source: Front Genet. 2024 Jul 31;15:1442938. doi: 10.3389/fgene.2024.1442938 (PMC11322057; doi:10.3389/fgene.2024.1442938)
Supplement: Supplementary file 1 [file Table1.docx]

Supplemental Table 1. The basic information of SNPs

| **SNPs** | **Gene** | **Major Allele** | **Minor Allele** | **Chromosome Position** | **Region** | **Function** |
| --- | --- | --- | --- | --- | --- | --- |
| rs649216 | KIR2DL4 | C | T | chr19:54813180 | Exon 6 | synonymous mutation |
| rs1051456 | KIR2DL4 | C | G | chr19:54813180 | Exon 4 | missense mutation |
| rs34785252 | KIR2DL4 | C | A | chr19:54814361 | 3' UTR | Non-coding |
| rs9380142 | HLA-G | G | A | chr 6: 29831017 | 3’TUR | Non-coding |
| rs1063320 | HLA-G | C | G | chr6:29830972 | 3' UTR | Non-coding |
| rs1630185 | HLA-G | A | G | chr 6: 29827880 | Exon 1 | synonymous mutation |
| rs1130363 | HLA-G | G | A | chr 6: 29829919 | Exon 5 | missense mutation |

Supplemental Table 2. Primers of SNPs

| **SNPs** | **Amplification** | | | |  | **Extension** | |
| --- | --- | --- | --- | --- | --- | --- | --- |
|  | **Forward primer** | **Reverse primer** | **Tm（℃）** | **Product （bp）** |  | **Primer** | **Direction** |
| rs649216 | CCTGGCAACCAAGAAATGAGAG | TCACAATCAGGCAACGGTCT | 60 | 365 |  | TTTTTTTTTTTTTTTTTTTTTTTTTTTTTTTTTGGAGAAAGAAGGGAAGGATGGT | R |
| rs1051456 | AGATCAGCAAGGGTGCACTG | ACCTGTGACAGAAACAGGCAG | 60 | 499 |  | TTTTTTTTTTTTTTTTTTTTTTGATCTCCCTACGAGTGGTCAGAC | F |
| rs34785252 | CTGTCTCTTGCTTACCAATGTCT | TATAAGGTTTGCTGATGCCAGA | 58 | 294 |  | TTTTTTTTTTTTTTTTTTTTTTTTTTTTCCAAACATACAAGAGGCTCTCT | F |
| rs9380142 | GAGTGGCAAGTCCCTTTGT | GGGAAGAGGTGTAGGGGTCT | 59 | 599 |  | TTTTTTTTACTTACTTCTGTATTAAAATTAGAATCTGAGT | F |
| rs1063320 | GAGTGGCAAGTCCCTTTGT | GGGAAGAGGTGTAGGGGTCT | 59 | 599 |  | TTTTTTTTTTTTTTTTTTAATACAGAAGTAAGTTATAGCTCAGTG | R |
| rs1630185 | TCCTGGTTCTAAAGTCCTCGC | TGTCGAACCGCACGAACT | 60 | 370 |  | TTTTTTTTTTTTTTTTTTTTTTTTTTTTTTTCCCGAACCCTCTTCCTGCT | F |
| rs1130363 | CCACCACCCTGTCTTTGACT | GCAGGGCACACTTCTACCTG | 60 | 573 |  | TTTTTTTTTTTTCCTTCCTTACCTGAGCTCTTCTT | R |

Supplemental Table 3. Hardy-Weinberg equilibrium test for each SNP in the control samples

| **SNPs** | **Gene** | **AA** | **AB** | **BB** | **Total** | **HWE-P** |
| --- | --- | --- | --- | --- | --- | --- |
| Mother |  |  |  |  |  |  |
| rs649216 | KIR2DL4 | 53(0.662) | 23(0.287) | 4(0.05) | 80 | 0.775 |
| rs1051456 | KIR2DL4 | 23(0.287) | 34(0.425) | 23(0.287) | 80 | 0.406 |
| rs34785252 | KIR2DL4 | 26(0.325) | 33(0.0.413) | 21(0.262) | 80 | 0.307 |
| rs9380142 | HLA-G | 24(0.3) | 32(0.4) | 24(0.3) | 80 | 0.201 |
| rs1063320 | HLA-G | 34(0.425 | 32(0.4) | 14(0.175) | 80 | 0.422 |
| rs1630185 | HLA-G | 27(0.337) | 40(0.5) | 13(0.162) | 80 | 0.96 |
| rs1130363 | HLA-G | 27(0.337) | 40(0.5) | 13(0.162) | 80 | 0.96 |
| Father |  |  |  |  |  |  |
| rs9380142 | HLA-G | 37(0.474) | 30(0.384) | 11(0.141) | 78 | 0.493 |
| rs1063320 | HLA-G | 27(0.346) | 30(0.384) | 21(0.269) | 78 | 0.135 |
| rs1630185 | HLA-G | 28(0.358) | 41(0.525) | 9(0.115) | 78 | 0.583 |
| rs1130363 | HLA-G | 28(0.358) | 41(0.525) | 9(0.115) | 78 | 0.583 |
| Offspring |  |  |  |  |  |  |
| rs9380142 | HLA-G | 34(0.425) | 30(0.375) | 16(0.200) | 80 | 0.171 |
| rs1063320 | HLA-G | 37(0.462) | 29(0.362) | 14(0.175) | 80 | 0.172 |
| rs1630185 | HLA-G | 20(0.25) | 45(0.562) | 15(0.187) | 80 | 0.511 |
| rs1130363 | HLA-G | 27(0.337) | 38(0.475) | 15(0.187) | 80 | 0.968 |

A：Major allele, B: minor allele

Supplemental Table 4. The genotype distribution of rs9380142 and rs1063320 loci within preeclampsia triads and controls triads

| **Preeclampsia** | | | | | |  | **Control** | | | | | |
| --- | --- | --- | --- | --- | --- | --- | --- | --- | --- | --- | --- | --- |
| **Mother** | **Father** | **Offspring** | | | **Total** |  | **Mother** | **Father** | **Offspring** | | | **Total** |
| rs9380142 | | AA | GA | GG |  |  | rs9380142 | | AA | GA | GG |  |
| AA | AA | 5 | 0 | 0 | 5 |  | AA | AA | 13 | 0 | 0 | 13 |
| AA | GA | 1 | 4 | 0 | 5 |  | AA | GA | 2 | 1 | 0 | 3 |
| GA | AA | 1 | 7 | 0 | 8 |  | GA | AA | 1 | 5 | 0 | 6 |
| GA | GA | 2 | 2 | 0 | 4 |  | GA | GA | 1 | 0 | 3 | 4 |
| AA | GG | 0 | 5 | 0 | 5 |  | AA | GG | 0 | 8 | 0 | 8 |
| GG | AA | 0 | 11 | 0 | 11 |  | GG | AA | 0 | 15 | 0 | 15 |
| GG | GA | 0 | 0 | 1 | 1 |  | GG | GA | 0 | 1 | 10 | 11 |
| GA | GG | 0 | 2 | 5 | 7 |  | GA | GG | 0 | 2 | 6 | 8 |
| GG | GG | 0 | 0 | 10 | 10 |  | GG | GG | 0 | 0 | 10 | 10 |
| Total |  | 9 | 31 | 16 | 56 |  | Total |  | 17 | 32 | 29 | 78 |
| rs1063320 | | CC | GC | GG |  |  | rs1063320 | | CC | GC | GG |  |
| CC | CC | 7 | 0 | 0 | 7 |  | CC | CC | 15 | 0 | 0 | 15 |
| CC | GC | 5 | 6 | 0 | 11 |  | CC | GC | 9 | 10 | 0 | 19 |
| GC | CC | 6 | 7 | 0 | 13 |  | GC | CC | 5 | 2 | 0 | 7 |
| GC | GC | 0 | 2 | 4 | 6 |  | GC | GC | 4 | 4 | 1 | 9 |
| CC | GG | 0 | 5 | 0 | 5 |  | CC | GG | 0 | 4 | 0 | 4 |
| GG | CC | 0 | 6 | 0 | 6 |  | GG | CC | 0 | 7 | 0 | 7 |
| GG | GC | 0 | 1 | 0 | 1 |  | GG | GC | 0 | 1 | 5 | 6 |
| GC | GG | 0 | 0 | 1 | 1 |  | GC | GG | 0 | 2 | 5 | 7 |
| GG | GG | 0 | 0 | 6 | 6 |  | GG | GG | 0 | 0 | 4 | 4 |
| Total |  | 18 | 27 | 11 | 56 |  | Total |  | 33 | 30 | 15 | 78 |
